# Direct and indirect effects of influenza vaccination

**DOI:** 10.1186/s12879-017-2399-4

**Published:** 2017-04-26

**Authors:** Martin Eichner, Markus Schwehm, Linda Eichner, Laetitia Gerlier

**Affiliations:** 1 Epimos GmbH, Dusslingen, Germany; 2 Institute for Clinical Epidemiology and Applied Biometry, University of Tübingen, Tübingen, Germany; 3 ExploSYS GmbH, Leinfelden, Germany; 4 QuintilesIMS, Real-World Evidence Solutions, Zaventem, Belgium

## Abstract

**Background:**

After vaccination, vaccinees acquire some protection against infection and/or disease. Vaccination, therefore, reduces the number of infections in the population. Due to this herd protection, not everybody needs to be vaccinated to prevent infections from spreading.

**Methods:**

We quantify direct and indirect effects of influenza vaccination examining the standard Susceptible-Infected-Recovered (SIR) and Susceptible-Infected-Recovered-Susceptible (SIRS) model as well as simulation results of a sophisticated simulation tool which allows for seasonal transmission of four influenza strains in a population with realistic demography and age-dependent contact patterns.

**Results:**

As shown analytically for the simple SIR and SIRS transmission models, indirect vaccination effects are bigger than direct ones if the effective reproduction number of disease transmission is close to the critical value of 1. Simulation results for 20–60% vaccination with live influenza vaccine of 2–17 year old children in Germany, averaged over 10 years (2017–26), confirm this result: four to seven times as many influenza cases are prevented among non-vaccinated individuals as among vaccinees. For complications like death due to influenza which occur much more frequently in the unvaccinated elderly than in the vaccination target group of children, indirect benefits can surpass direct ones by a factor of 20 or even more than 30.

**Conclusions:**

The true effect of vaccination can be much bigger than what would be expected by only looking at vaccination coverage and vaccine efficacy.

**Electronic supplementary material:**

The online version of this article (doi:10.1186/s12879-017-2399-4) contains supplementary material, which is available to authorized users.

## Background

After vaccination, vaccinees acquire some protection against infection and/or disease. As successfully vaccinated individuals cannot be infected, they also cannot pass on the infection to others. Thus, the infection probability drops for unprotected individuals as well. Due to this indirect effect, called “herd protection”, not everybody needs to be vaccinated to prevent infections from spreading. For influenza, such indirect protection effects have been demonstrated in several studies: in the US, vaccination of 20–25% of children (2–18 years) reduced adults’ physician consultations for respiratory illness by up to 18% [1]. In Canada, vaccination of 83% of children (<=15 years) reduced influenza infection incidence in unvaccinated individuals by 61% [2]. In Japan, vaccination of school-age children reduced influenza mortality among the elderly [3]. In the UK, vaccination of children significantly reduced influenza-related medical resource use in adults [4]. Supplementing these real-world observations, simulation studies on influenza frequently reported strong indirect effects: even relatively low vaccine coverage rates have been shown to yield important public health benefits in the US [5]. Indirect effects can even exceed direct effects [6]. Modeling studies of pediatric vaccination in the UK predicted more indirect than direct effects [7]. Disentangling indirect vaccination benefits remains challenging [8] as also the vaccination target group and even vaccinees who did not become immune or who lost their immunity benefit from herd effects. The aim of this study is to explain and quantify direct and indirect influenza vaccination effects. We approach this issue by studying two simple mathematical models and by running and analyzing computer simulations on influenza vaccination in Germany.

## Methods and results

### Direct and indirect effects in the SIR model

**Fig. 1:**
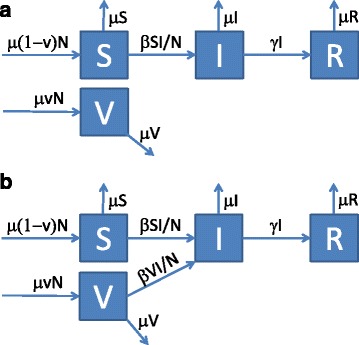
**a**-**b** SIR model describing the transmission of infection in a population (S: susceptible, I: infectious, R: immune, V vaccinated). **a** Standard SIR model where a fraction v is vaccinated at birth and immediately becomes immune. **b** Modified SIR model with vaccinees who can become infected. Parameters: per capita birth and death rate μ, contact rate β, recovery rate γ, population size N. The full model description is given in the Additional file 1

**Fig. 2:**
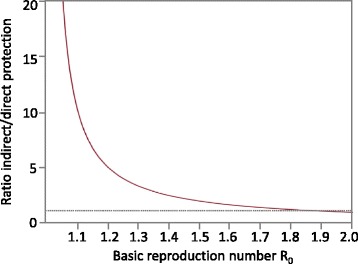
Ratio of indirect/direct vaccination effects in the SIR model. This ratio does not depend on the vaccination coverage as long as it does not completely prevent the spread of infection

The classic susceptible-infected-recovered (SIR) model forms the backbone of most infectious disease transmission models (Fig. 1a). Vaccinated newborns are assumed to become fully immune (V) whereas unvaccinated ones are susceptible (S). These can become infected (I) after contact with infected individuals; they finally recover, and become permanently immune (R). Although for influenza, neither vaccination-derived nor naturally acquired immunity lasts lifelong (as is assumed in the SIR model), we start with analyzing this basic model for the sake of simplicity. In order to quantify direct and indirect effects of vaccinations (which in this model only occur shortly after birth), we have slightly re-structured the SIR model: Fig. 1a shows a version of the standard model (model A) where vaccinated individuals (V) are fully immune. Figure 1b shows a version where vaccinees are fully susceptible to infection (i.e. vaccination in Fig. 1b is assumed to be completely useless); model B, therefore, represents the SIR model without vaccination effects. It shows how many vaccinees and how many non-vaccinees would have been infected if the vaccination either had never occurred or if it were completely useless. In the standard SIR model A, infection incidence is reduced because vaccinees are immune: they can neither be infected nor can they cause secondary infections. As a consequence, fewer susceptible individuals (S) are infected in model A than in the modified model B where the vaccination is without effect. In a first step, we only use model A to calculate the infection incidence for two vaccination scenarios: I_0_ is the infection incidence without any vaccinations and I_S_ is the incidence with vaccinations. The total effect of vaccination (comprising both, direct and indirect effects) is given by the difference I_0_-I_S_. In a second step, we take a look at model (b) where vaccination is regarded to be non-protective: as vaccinees can be infected, we can calculate their infection incidence I_V_, too. As these infections will not occur if the vaccine is fully protective, I_V_ is the direct effect of vaccination. The indirect effect is finally given as difference between total and direct effect (I_0_-I_S_-I_V_). For the SIR model, the evaluation can be done by looking at the equilibrium state of the corresponding set of differential equations (as given in the Additional file 1). A key parameter in the calculation is the so-called basic reproduction number R_0_ which describes how many secondary infections are caused by a single initial case in a completely non-immune population without interventions. Calculating the ratio of indirect/direct protection, we obtain the expression 1/(R_0_–1) which is displayed in Fig. 2. If R_0_ is larger than 2 (as is the case for e.g. measles), indirect effects are smaller than direct ones (the ratio becomes less than 1); if R_0_ is below 2 (as is the case for influenza), indirect effects exceed direct ones.

### Direct and indirect effects in the SIRS model

**Fig. 3:**
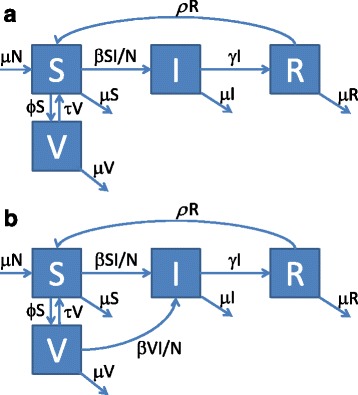
**a**-**b** SIRS model describing the transmission of infection in a population (S: susceptible, I: infectious, R: immune, V vaccinated). **a** Standard SIRS model with protective vaccination. **b** Modified SIRS model with vaccinees who can become infected. Parameters: per capita birth and death rate μ, contact rate β, recovery rate γ, vaccination rate ϕ, loss rate of naturally acquired immunity *ρ*, loss rate of vaccination-derived immunity τ, population size N. The full model description is given in the Additional file 1

**Fig. 4:**
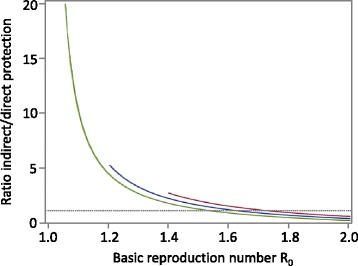
Ratio of indirect/direct vaccination effects in the SIRS model for different vaccination rates (from top to bottom: ϕ = 0.2, 0.1, 0.01 per year). As the whole population is eligible for vaccination in the SIRS model, transmission can go to extinction for moderately high annual vaccination coverage if R_0_ is small (thus, the lines cannot be drawn for the whole R_0_ range). Parameters: life expectancy μ^−1^ = 70 years, duration of naturally acquired immunity *ρ* ^*−1*^ = 6 years, duration of vaccination-derived immunity τ^−1^ = 2 years, duration of contagiousness γ^−1^ = 5 days. The mathematical description of the curves is given in the Additional file 1

The SIRS extension (Fig. 3a) of the SIR model allows for loss of immunity and for repeated vaccinations which not necessarily need to occur shortly after birth. Thus, it represents the situation of influenza much better than the SIR model. Individuals are born susceptible (S); they can either be vaccinated (V) or they can become infected (I) after contact with infected individuals and finally recover, and become immune (R). Immune individuals can lose their immunity and become susceptible again (S) whereby the immunity loss rate can be different for naturally acquired (R) and vaccination-derived immunity (V). We again modified the model: Fig. 3a shows the standard SIRS model where vaccinated individuals (V) are immune; Fig. 3b shows the version where vaccinees remain fully susceptible. Describing the systems by differential equations again allows calculating the ratio of indirect/direct vaccination effects as outlined in the previous section (see Additional file 1 for details). As shown in Fig. 4, the ratio becomes big if the basic reproduction number R0 is small.

### Direct and indirect effects in seasonal transmission models

Seasonality is neither considered in the basic SIR nor in the SIRS model, yet it plays a major role in the transmission of influenza. More realistic simulation models frequently implement a seasonality module which compresses the main influenza transmission period to a few winter months [7, 9–13] (for a closer look at the specific effects of seasonality on direct and indirect effects, see Additional file 1). In Germany, influenza vaccination is mainly recommended for the elderly and for other individuals at increased risk whereby mostly trivalent inactivated vaccine is used. Following a general discussion on pediatric influenza vaccination, a survey initiated by the Robert-Koch-Institute in 2015 revealed that over 50% of parents are willing to have their children vaccinated against influenza if it is officially recommended [14]. In the following, we use an extension of the previously published influenza transmission model QLAIV-Sim [11, 12, 15] to look at direct and indirect effects of pediatric influenza vaccination in Germany. QLAIV-Sim is based on a model which extends the SIRS model to a system of 32,330 differential equations: based on realistic demographic data [16], the population is structured in 101 age groups, further distinguished into “at risk” and “no risk” groups, who are connected by an age-dependent contact matrix [17]. Vaccinations, using reported age- and risk-dependent vaccination coverage [18, 19], occur annually in October and November whereby individuals who were vaccinated in the previous season are preferentially vaccinated again (see Additional file 1 for details [20]). Vaccine efficacy of the inactivated vaccine is assumed to be 45% (0.5–1 year old children), 59% (2–17), 60% (18–59 no risk individuals) and 58% (at risk individuals and elderly 60+) [21–24]; the vaccine efficacy of the live vaccine for 2–17 year old children is assumed to be 80% in the season following vaccination and 56% in the subsequent season [24, 25]. In order to create realistic age-dependent immunity patterns in the population, the model is run from 2000 to 2016 with trivalent influenza vaccine (TIV), using the recorded vaccine composition and allowing for the independent transmission of the four influenza strains A(H1N1), A(H3N2), B/Yamagata and B/Victoria. During the following 10-year evaluation period (starting in 2017), only quadrivalent influenza vaccines are used (i.e. vaccines which contain both Influenza B lineages). In a first simulation, we calculate how many infections occur (“baseline” result) if vaccinations are performed as in the initialization period with the only exception, that quadrivalent inactivated vaccine (QIV) is used instead of TIV. In a second step, we run the same simulation with the identical vaccination coverage, except that an increased percentage of children (2–17 years) receive quadrivalent live vaccine (QLAIV) instead of QIV. The resulting difference in infection incidence is the total effect of the additional QLAIV vaccinations. In order to separate this total effect into direct and indirect effects, we use the same strategy as described above: a third simulation is run where we assume that QLAIV vaccination is completely without effect, and the cumulative incidence is recorded separately for individuals who are in stage S and in stage V, respectively.

**Fig. 5:**
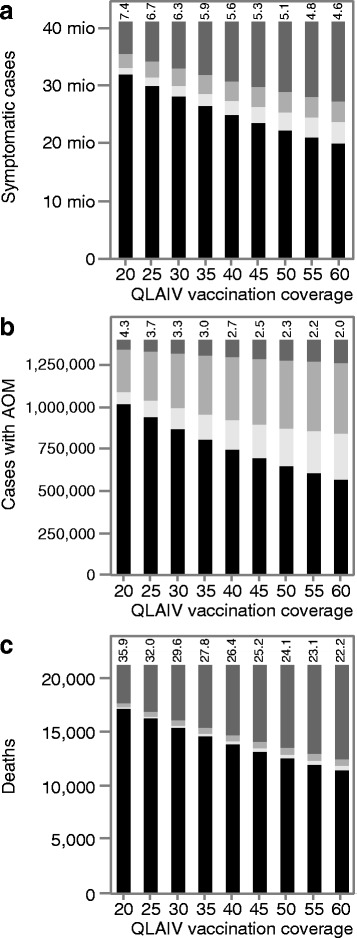
**a**-**c** Simulation results of pediatric QLAIV vaccination in Germany. Each bar represents the results for 10 years (2017–26): dark grey: indirectly prevented cases among adults, light grey: indirectly prevented cases among children, white: directly prevented cases among children, black: remaining cases which are not prevented. Numbers above the bars give the ratios “all indirectly prevented cases” / “all directly prevented cases”. Simulations are initialized from 2000 to 2016 using TIV with the baseline vaccination coverage. In the 10-year period starting with 2017, vaccinations are switched to QIV (reference scenario) and the effect of additional QLAIV vaccination of 2–17 years old children is evaluated. In the QLAIV scenario, children below 2 and adults receive QIV as in the reference scenario; in the first evaluation year the QLAIV coverage of 2–17 year old children is identical to the baseline coverage (around 5%), then it is increased stepwise for 3 years to reach a final coverage of 20 to 60%. **a** Symptomatic cases; **b** cases with acute otitis media (AOM; percentages of symptomatic cases in the “no risk” group: 0–1 year: 39.7%, 2–6: 19.6%, 7–12: 4.4%, 13–17: 4%, 18+: 1%; in the risk group: 1% [32–35]), (**c**) deaths due to influenza (percentages of symptomatic cases in the “no risk” group: 0–1 year: 0.062%, 2–6: 0.027%, 7–12. 0.011%, 13–17: 0.005%, 18+: 0.0132%; in the risk group: 0.13%, guided by [36, 37]). For numerical results, see Additional file 1: Table S3

Table 1 shows the simulation results for 20% annual QLAIV vaccination coverage; infections are translated into symptomatic cases, assuming that 66.9% of infections lead to clinical disease [26]. Without additional vaccinations, 40.9 million symptomatic cases are expected to occur in the 10-year evaluation period. QLAIV vaccination of 20% of children reduces the cumulative incidence to 31.9 million cases. The prevented 9 million cases can be split into 1.1 million directly prevented cases among vaccinees, 2.4 million indirectly prevented cases among non-vaccinated children and 5.5 million indirectly prevented cases among adults. Thus, more than twice as many cases among children are indirectly prevented than directly. Combining all indirectly prevented cases among children and adults (7.9 million), we can see that more than 7 times as many cases are indirectly prevented as are directly prevented. Fig. 5a shows the results for 20 to 60% annual QLAIV coverage: the number of directly prevented symptomatic cases (white) and the numbers of indirectly prevented ones (light and dark grey) increase with increasing QLAIV coverage; due to mathematical reasons, the ratio of indirectly/directly prevented cases declines from 7.4 to 4.6 with increasing coverage. The numbers of symptomatic cases are finally translated into cases of acute otitis media (AOM) and to deaths due to influenza (Fig. 5b-c). As AOM cases predominantly occur among children, more cases are (directly and indirectly) prevented among children, leading to lower ratios of indirectly/directly prevented cases (ranging from 2.0 to 4.3; Fig. 5b). Considering, on the other hand, influenza deaths which predominantly occur in the elderly, the ratios of indirectly/directly prevented cases become much higher (ranging from 22.2 to 35.9; Fig. 5c), meaning that for every directly prevented death among vaccinees up to 36 deaths are indirectly prevented. In a sensitivity analysis, the vaccine efficacy of QLAIV is reduced to the QIV value of 59% and the duration of QLAIV immunity is reduced to 1 year (as for QIV), the absolute vaccination effects are reduced accordingly, but the ratio of indirect / direct effects grows: for 20% to 60% vaccination coverage, the ranges of ratios are 5.1–10.1 (symptomatic cases), 2.5–8.6 (AOM) and 23.4–63.0 (deaths), respectively (full results are shown in Additional file 1: Table S4).

**Table 1:** Simulated number of symptomatic cases (pooled over 10 years, 2017–26) for children, for adults and for the total German population

Age group	Symptomatic cases over 10 years			Effects: direct effect in children d_C_ = I_V_ indirect effects I_0_ - (I_S_ + I_V_) in children (i_C_), adults (i_A_) and the total population (i_T_)
	Sim. 1: I_0_ (no additional vaccinations)	Sim. 2: I_S_ (with additional protective QLAIV vaccinations)	Sim. 3: I_V_ (with additional non-protective vaccinations)	
Children (target group)	10,315,818	6,826,372	1,080,655	d_C_ = 1,080,655 i_C_ = 2,408,791 ratio for children only: i_C_ / d_C_ = 2.2
Adults (non-target group)	30,560,738	25,024,812	0	i_A_ = 5,535,926
Total population	40,876,556	31,851,184	1,080,655	d_C_ = 1,080,655 i_T_ = 7,944,717 ratio for total population: i_T_ / d_C_ = 7.4

The 2nd column shows the results if QIV is used with unchanged baseline vaccination coverage (reference scenario). The 3rd column shows what happens if 20% QLAIV vaccination of 2–17 year old children is used in addition to the baseline QIV coverage of other age-grous. The 4th column shows what happens if the same strategy is used as in the 3rd column with a non-protective vaccine instead of QLAIV. The 5th column shows the calculations of direct and indirect effects and of ratios. For numerical results with different QLAIV coverage, see Additional file 1: Table S3

## Discussions

It is well-known that vaccination not only protects vaccinated individuals, but also causes indirect effects which often are called “herd effects” or “herd immunity”. Indirect effects are assumed to provide a little additional benefit, but the huge effects for influenza which are shown in Fig. 5a-c surpass all expectations of a “little benefit”. As has been shown for the simple SIR and SIRS models (Figs. 2 and 4), huge indirect effects are not restricted to sophisticated simulation tools, but are a common feature of transmission models, particularly if the reproduction number is close to the critical value of 1.0 for which infections can no longer circulate. For measles with a frequently quoted basic reproduction number R_0_ = 15 [27, 28], such effects would only be observed if the initial immunity already surpassed 90% and if additional vaccinations would further reduce transmission. For influenza, estimates of R_0_ are much closer to 1 than for measles [29]. Calibration of QLAIV-Sim to the observed annual infection incidence of 10.6% among young adults in Germany [30] led to all-year average of R_0_ = 1.1. Because of seasonal fluctuations in transmissibility [9], the time dependent magnitude of R_0_ fluctuates from 0.63 (in summer) to 1.57 (around Christmas) in Germany. Due to baseline vaccinations and to previous infections, about 30% of the population is immune at the beginning of the annual transmission season. Thus, an infectious individual cannot infect R_0_, but only R_e_ = (1–0.3)^.^R_0_ others (this is called the effective reproduction number). Even at the seasonal peak, R_e_(t) only reaches a value of (1–0.3)^.^1.57 = 1.1, which is so close to the critical value 1, that huge indirect effects must occur if the immunity in the population is further increased by additional vaccinations.

At first glance, it may seem counter-intuitive that the ratio of indirect vs. direct effects decreases if the percentage of immunized children grows. This is mainly due to saturation and competition effects. The shift towards lower ratios for high vaccination coverage can most easily be explained by imagining a very large coverage which immunizes so many individuals that the direct effect itself becomes so large that applying the highest indirect/direct ratios (>30, in Fig. 5) would necessitate that more cases have to be prevented indirectly than occur without vaccination. This also explains why the indirect/direct ratios become even bigger if a lower vaccine efficacy and a lower duration of protection is used for QLAIV (Additional file 1: Table S4) because this corresponds to a lower percentage of children who are immunized (i.e. a result which could also be achieved by using a lower vaccination coverage).

**Fig. 6:**
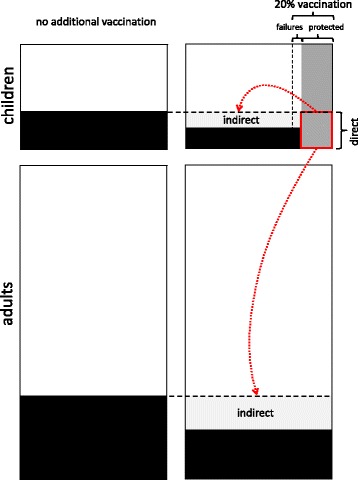
Schematic figure of direct and indirect effects of pediatric vaccination. The upper boxes depict children, the lower ones adults. Boxes on the left show the baseline situation without additional vaccinations; on the right, 20% of children is vaccinated with a vaccine efficacy of 80%. Black areas show infected individuals, dark grey areas show vaccinated individuals. The shaded part of the vaccinated individuals depicts children who would have been infected and are, thus, directly protected. Preventing these cases also reduces the infection rate for unvaccinated children and for adults, causing indirect effects depicted in light grey

In pilot vaccination areas in England 2014/15, 58.6% of children (ages 4–11) were given QLAIV vaccination. This prevented over 90% of doctoral visits due to ILI of 5–10 year old children (i.e. the target population) and about half of the doctoral visits of adults [31]. Using 60% coverage (ages 2–17) in our simulations prevents 70% of all symptomatic cases among children and 48% of all adult cases, indicating that our simulation results for Germany are slightly less optimistic than the field data from England. Comparing the number of prevented cases in the non-target population with the number of prevented cases in the target population gives a first indication of the indirect vaccination effect, yet it strongly under-estimates the true indirect/direct protection ratio. The POLYMOD study has shown that individuals of all ages, but especially children and juveniles, have most of their contacts with others who are of similar age [17]. Consequently, non-vaccinated children also benefit strongly if other children are vaccinated and cannot pass on the infection to them, as is shown schematically in Fig. 6 using 20% QLAIV coverage: more than twice as many cases are prevented among non-vaccinated children as among vaccinated ones (Table 1). This indirect effect among children has to be added to the indirect effect among adults to obtain the full indirect effect of vaccination. Comparing all indirectly and all directly prevented cases in Table 1 leads to an indirect/direct ratio of 7.4 for 20% QLAIV coverage, but the ratio of prevented adult cases/prevented children cases is only 1.6 (5.5 million adult vs. 3.5 million children cases). If an area without vaccination is compared with a spatially separate area with vaccination (as was the case with the English pilot study mentioned above), the expected number of directly prevented cases per 100,000 individuals can easily be calculated as the product of (1) the incidence per 100,000 individuals of the target age-group in the control area, (2) the vaccinated fraction of the target age-group in the vaccination area, and (3) the vaccine efficacy (cf. Fig. 6). For the target age-group, the observed case difference between control and vaccinated area should be bigger than the expected direct effect, as indirect effects also occur. For all non-target age-groups, the differences between control and vaccinated area must also be attributed to indirect effects. These findings should be used to further encourage vaccinations in order to maximize the direct and indirect effects in the population.

If the effective reproduction number of disease transmission is low, the true effect of vaccination campaigns can be much bigger than what would be expected by only looking at vaccination coverage and vaccine efficacy: for influenza, four to seven times as many cases can be prevented among non-vaccinated individuals as among vaccinees. If disease-related complications occur more frequently in the unvaccinated age-groups than in the vaccinated ones, indirect benefits can surpass direct ones by a factor of 20 or even more than 30 (Fig. 5a-c).

## Additional file

Additional file 1: Contains detailed mathematical descriptions of the SIR model, the SIRS model and the Q-LAIV-Sim simulation model. It furthermore provides details on the calculation of direct and indirect effects an on the obtained results. For the simulation model, the influence of seasonality on indirect effects is explored. (PDF 319 kb).

## Abbreviations

AOM: Acute otitis media
QIV: Quadrivalent inactivated influenza vaccine
QLAIV: Quadrivalent live influenza vaccine
R_0_: Basic reproduction number
R_e_: Effective reproduction number
SIR: Susceptible-Infectious-Recovered model of pathogen transmission
SIRS: Susceptible-Infectious-Recovered-Susceptible model of pathogen transmission
TIV: Trivalent inactivated influenza vaccine
