# Supplementary material for: Direct and indirect effects of influenza vaccination
Source: BMC Infect Dis. 2017 Apr 26;17:308. doi: 10.1186/s12879-017-2399-4 (PMC5405516; doi:10.1186/s12879-017-2399-4)
Supplement: Additional file 1: — Contains detailed mathematical descriptions of the SIR model, the SIRS model and the Q-LAIV-Sim simulation model. It furthermore provides details on the calculation of direct and indirect effects an on the obtained results. For the simulation model, the influence of seasonality on indirect effects is explored. (PDF 319 kb). [file 12879_2017_2399_MOESM1_ESM.pdf]

Online supporting material for "Direct and indirect effects of influenza vaccination"

Martin Eichner, Markus Schwehm, Linda Eichner, Laetitia Gerlier

Exploring direct and indirect vaccination effects in the SIR model

The susceptible-infected-recovered (SIR) model forms the backbone of most infectious disease transmission models. Unvaccinated newborn babies are susceptible (S), become infected (I) after having had contact with infected individuals and finally recover (R) to acquire lifelong immunity (parameters: per capita birth and death rate μ, contact rate β, recovery rate γ, population size N). The system is frequently described by the following set of differential equations:

$$dS/dt = \mu(1-v)N - \beta SI/N - \mu S$$
$$dI/dt = \beta SI/N - (\gamma+\mu)I$$
$$dR/dt = \gamma I - \mu R$$
$$dV/dt = \mu v N - \mu V$$

The endemic equilibrium of this system is given by

$$S^* = N/R_0 \quad \text{with} \quad R_0 = \beta/(\gamma+\mu)$$
$$I^* = \mu N((1-v)R_0 - 1)/\beta$$
$$V^* = vN$$
$$R^* = N - S^* - I^* - V^*$$

A modified SIR model with non-protective vaccination can, accordingly, be written as

$$d\bar{S}/dt = \mu(1-v)N - \beta\bar{S}\bar{I}/N - \mu\bar{S}$$
$$d\bar{I}/dt = \beta\bar{S}\bar{I}/N - (\gamma+\mu)\bar{I}$$
$$d\bar{R}/dt = \gamma\bar{I} - \mu\bar{R}$$
$$d\bar{V}/dt = \mu v N - \beta\bar{V}\bar{I}/N - \mu\bar{V}$$

The endemic equilibrium of this system is given by

$$\bar{S}^* = (1-v)N/R_0$$
$$\bar{I}^* = \mu N(R_0 - 1)/\beta$$
$$\bar{V}^* = vN/R_0$$
$$\bar{R}^* = N - \bar{S}^* - \bar{I}^* - \bar{V}^*$$

The main purpose of the second model is to provide the infection incidence for unvaccinated individuals which is not influenced by indirect effects to determine how many non-vaccinees would be infected in the absence of indirect effects. Evaluating only the first model, the equilibrium incidence of infection is given by $\beta S^* I^* / N$; the infection incidence is reduced by vaccination (still only using the first model); it the infection is not completely eliminated by the vaccination, the reduction is given by $\frac{\beta N}{R_0 N}\cdot\frac{\mu N(R_0-1)}{\beta} - \frac{\beta N}{R_0 N}\cdot\frac{\mu N((1-v)R_0-1)}{\beta} = \mu v N$. The second model allows calculating what the infection incidence among vaccinees alone would have been:

$\frac{\beta\bar{V}^*\bar{I}^*}{N} = \frac{\beta}{N}\cdot\frac{vN}{R_0}\cdot\frac{\mu N(R_0-1)}{\beta} = \mu v N\left(1-\frac{1}{R_0}\right)$. With a protective vaccine, none of these infections occur; thus, these infections are directly prevented by vaccination with a protective vaccine. The difference between the total effect and the direct effect, $\mu v N - \mu v N\left(1-\frac{1}{R_0}\right) = \frac{\mu v N}{R_0}$, is the indirect effect, and the ratio of indirect/direct effect is

$\frac{\mu v N}{R_0}\Big/\left(\mu v N\left(1-\frac{1}{R_0}\right)\right) = \frac{1}{R_0 - 1}$. Indirect effects surpass direct ones for $1 < R_0 < 2$ (cf. Figure 2 of the paper). The all-year average R_0=1.1 which has been used in the influenza simulations with Q-LAIV-Sim translates into a ratio of 10, suggesting, that indirect effects should surpass direct ones by a factor of 10.

Exploring direct and indirect vaccination effects in the SIRS model

As the immunity against influenza is not permanent and as vaccinations have to be given repeatedly, the simple SIR model does not adequately model the transmission of influenza. The so-called SIRS model improves some of the shortcomings of the SIR model: immunity is lost over time and vaccination is modeled as a continuous process which goes on throughout the life. As in the previous section, we develop a second SIRS model where vaccination does not prevent infection in order to separate direct and indirect vaccination effects (parameters: per capita birth and death rate μ, contact rate β, recovery rate γ, vaccination rate ϕ, loss rate of naturally acquired immunity ρ, loss rate of vaccination-derived immunity τ, population size N). The SIRS models can be described by the following sets of differential equations:

$$\frac{dS}{dt} = \mu N + \rho R + \tau V - \frac{\beta SI}{N} - (\phi + \mu)S$$
$$\frac{dI}{dt} = \frac{\beta SI}{N} - (\gamma + \mu)I$$
$$\frac{dR}{dt} = \gamma I - (\rho + \mu)R$$
$$\frac{dV}{dt} = \phi S - (\tau + \mu)V$$

The endemic equilibrium of this system is given by

$$S^* = \frac{N}{R_0} \quad \text{with} \quad R_0 = \frac{\beta}{\gamma + \mu}$$
$$I^* = N\left(\left(1 - \frac{1}{R_0}\right) - \frac{\phi}{\mu + \tau}\right)\frac{\mu + \rho}{\gamma + \mu + \rho}$$
$$V^* = \frac{N\phi}{R_0(\mu + \tau)}$$
$$R^* = N - S^* - I^* - V^*$$

Similarly, the modified SIR model with non-protective vaccination is given by

$$\frac{d\bar{S}}{dt} = \mu N + \rho \bar{R} + \tau \bar{V} - \frac{\beta \bar{S}\bar{I}}{N} - (\phi + \mu)\bar{S}$$
$$\frac{d\bar{I}}{dt} = \frac{\beta(\bar{S} + \bar{V})\bar{I}}{N} - (\gamma + \mu)\bar{I}$$
$$\frac{d\bar{R}}{dt} = \gamma \bar{I} - (\rho + \mu)\bar{R}$$
$$\frac{d\bar{V}}{dt} = \phi \bar{S} - \frac{\beta \bar{V}\bar{I}}{N} - (\tau + \mu)\bar{V}$$

The endemic equilibrium of this system is given by

$$\bar{S}^* = \frac{N}{R_0}\left(1 - \frac{(\gamma + \rho + \mu)\phi}{R_0(\gamma + \mu)(\rho + \mu) + (\gamma + \rho + \mu)(\tau + \phi) - \gamma\rho}\right)$$
$$\bar{I}^* = N\left(1 - \frac{1}{R_0}\right)\frac{\rho + \mu}{\gamma + \rho + \mu}$$
$$\bar{V}^* = \frac{N}{R_0} \cdot \frac{(\gamma + \rho + \mu)\phi}{R_0(\gamma + \mu)(\rho + \mu) + (\gamma + \rho + \mu)(\tau + \phi) - \gamma\rho}$$
$$\bar{R}^* = N - \bar{S}^* - \bar{I}^* - \bar{V}^*$$

The main purpose of the second model is to provide the infection incidence for unvaccinated individuals which is not influenced by indirect effects to determine how many non-vaccinees would be infected in the absence of indirect effects. Comparing the results of only the first model with and without vaccination, the incidence of infections which is prevented by vaccination totals $\frac{N\phi}{R_0} \cdot \frac{\gamma + \mu}{\gamma + \rho + \mu} \cdot \frac{\rho + \mu}{\tau + \mu}$. The incidence among vaccinees in the second model,

$$\frac{\beta \bar{V}^* \bar{I}^*}{N} = N\left(1 - \frac{1}{R_0}\right)\frac{\phi(\gamma + \mu)(\rho + \mu)}{R_0(\gamma + \mu)(\rho + \mu) + (\gamma + \rho + \mu)(\tau + \phi) - \gamma\rho}$$

, denotes infections that would have occurred among vaccinees (i.e. infections which are directly prevented by a protective vaccine). The difference between the total effect and the direct effect is the indirect effect. The ratio of indirect/direct effects is

$$\frac{N\left(1 - \frac{1}{R_0}\right)\frac{\phi(\gamma + \mu)(\rho + \mu)}{R_0(\gamma + \mu)(\rho + \mu) + (\gamma + \rho + \mu)(\tau + \phi) - \gamma\rho}}{\frac{N\phi}{R_0} \cdot \frac{\gamma + \mu}{\gamma + \rho + \mu} \cdot \frac{\rho + \mu}{\tau + \mu} - N\left(1 - \frac{1}{R_0}\right)\frac{\phi(\gamma + \mu)(\rho + \mu)}{R_0(\gamma + \mu)(\rho + \mu) + (\gamma + \rho + \mu)(\tau + \phi) - \gamma\rho}}$$

(cf. Figure 4 of the paper).

Exploring direct and indirect vaccination effects with the simulation tool Q-LAIV-Sim

The simulation tool Q-LAIV-Sim describes the spread of influenza in a population with realistic demographic structure, age-dependent contacts, annual vaccination and loss of immunity. Previous versions of the simulation tool have been published elsewhere (1, 2). The new version of the simulation tool differs in several aspects from the previously published one: (a) instead of calculating the demographic development, the demography in the new version of the simulation tool is solely based on demographic data and on official demographic predictions (for details, see paragraph "Birth ν, death μ and ageing α"). (b) Instead of using a single "birthday" at the beginning of the simulation year at which the age of every individual in the whole population increases by one year, ageing of the population now occurs continuously throughout the simulation year (for details, see paragraph "Birth ν, death μ and ageing α"). (c) The POLYMOD matrix which describes the age-dependent frequency of contacts was extended to 1-year age groups and made symmetric by averaging "incoming" and outgoing" contacts (for details, see paragraph "Contact matrix"). (d) Whereas the previous version of the simulation tool only distinguished between influenza A and B, the extended version allows for the independent transmission of four influenza viruses (A(H1N1), A(H3N2), B/Yamagata, B/Victoria; for details, see paragraph "Influenza strains"). (e) This also allows to consider that trivalent influenza vaccine (TIV) only protects against the B lineage contained in the vaccine (for details, see paragraph "Vaccination rate ϕ"). (f) Vaccination in the new version not only depends on the age and risk-status of the vaccinees, but also considers whether or not they were vaccinated in the previous season, allowing for preferential re-vaccination (the calculation of the re-vaccination factor from data will be given below). (g) Vaccination-derived immunity, which was assumed to be lost exponentially in the previous version, is now assumed to be lost completely at the end of one or two years (for details, see paragraph "Loss of vaccine-derived immunity"). (h) Introduction of infections from outside of the population (which was already considered in the previous version of the simulation tool) was modified to mainly occur during the main transmission season, assuming that neighboring countries (from which infections are most likely introduced) have similar seasonal waves (for details, see paragraph "Force of infection λ"). (i) Newer parameter values (e.g. those describing the vaccination coverage, the vaccine efficacy and the percentage of at-risk individuals) have become available and are now used in the simulations (Tables S1 and S2).

Model description of Q-LAIV-Sim

The core of the mathematical model which underlies the computer simulations is given below. In order to reduce the complexity of the model which is described by 32,330 differential equations, only the basic features are shown in the equations; further explanations will be given below.

Individuals with maternal protection *M*

$$\frac{dM_{a,n}}{dt} = -\omega M_{a,n} - \alpha_a(t)M_{a,n} - \mu_a(t)M_{a,n} + \begin{cases} m\,(1-r_a)\nu(t) & \text{if } a=0 \\ \alpha_{a-1}(t)\dfrac{1-r_a}{1-r_{a-1}}M_{a-1,n} & \text{else} \end{cases}$$

$$\frac{dM_{a,r}}{dt} = -\omega M_{a,r} - \alpha_a(t)M_{a,r} - \mu_a(t)M_{a,r} + \begin{cases} m\,r_a\nu(t) & \text{if } a=0 \\ \alpha_{a-1}(t)\left(M_{a-1,r} + \dfrac{r_a - r_{a-1}}{1-r_{a-1}}M_{a-1,n}\right) & \text{else} \end{cases}$$

Susceptible individuals *S*

$$\frac{dS_{a,n}}{dt} = \rho R_{a,n} + \omega M_{a,n} - \lambda_a(t)S_{a,n} - \phi_{a,n}(t)S_{a,n} - \alpha_a(t)S_{a,n} - \mu_a(t)S_{a,n} + \begin{cases} (1-m)(1-r_a)\nu(t) & \text{if } a=0 \\ \alpha_{a-1}(t)\dfrac{1-r_a}{1-r_{a-1}}S_{a-1,n} & \text{else} \end{cases}$$

$$\frac{dS_{a,r}}{dt} = \rho R_{a,r} + \omega M_{a,r} - \lambda_a(t)S_{a,r} - \phi_{a,r}(t)S_{a,r} - \alpha_a(t)S_{a,r} - \mu_a(t)S_{a,r} + \begin{cases} (1-m)r_a\nu(t) & \text{if } a=0 \\ \alpha_{a-1}(t)\left(S_{a-1,r} + \dfrac{r_a - r_{a-1}}{1-r_{a-1}}S_{a-1,n}\right) & \text{else} \end{cases}$$

Infected individuals who are not yet contagious *E*

$$\frac{dE_{a,n}}{dt} = \lambda_a(t)\left(S_{a,n} + (1-P)V_{a,n}\right) - \delta E_{a,n} - \alpha_a(t)E_{a,n} - \mu_a(t)E_{a,n} + \begin{cases} 0 & \text{if } a=0 \\ \alpha_{a-1}(t)\dfrac{1-r_a}{1-r_{a-1}}E_{a-1,n} & \text{else} \end{cases}$$

$$\frac{dE_{a,r}}{dt} = \lambda_a(t)\left(S_{a,r} + (1-P)V_{a,r}\right) - \delta E_{a,r} - \alpha_a(t)E_{a,r} - \mu_a(t)E_{a,r} + \begin{cases} 0 & \text{if } a=0 \\ \alpha_{a-1}(t)\left(E_{a-1,r} + \dfrac{r_a - r_{a-1}}{1-r_{a-1}}E_{a-1,n}\right) & \text{else} \end{cases}$$

Infected individuals who are contagious *I*

$$\frac{dI_{a,n}}{dt} = \delta E_{a,n} - \gamma I_{a,n} - \alpha_a(t)I_{a,n} - \mu_a(t)I_{a,n} + \begin{cases} 0 & \text{if } a=0 \\ \alpha_{a-1}(t)\dfrac{1-r_a}{1-r_{a-1}}I_{a-1,n} & \text{else} \end{cases}$$

$$\frac{dI_{a,r}}{dt} = \delta E_{a,r} - \gamma I_{a,r} - \alpha_a(t)I_{a,r} - \mu_a(t)I_{a,r} + \begin{cases} 0 & \text{if } a=0 \\ \alpha_{a-1}(t)\left(I_{a-1,r} + \dfrac{r_a - r_{a-1}}{1-r_{a-1}}I_{a-1,n}\right) & \text{else} \end{cases}$$

Individuals who are immune due to vaccination *V*

$$\frac{dV_{a,n}}{dt} = \phi_{a,n}(t)S_{a,n} - \lambda_a(1-P)V_{a,n} - \alpha_a(t)V_{a,n} - \mu_a(t)V_{a,n} + \begin{cases} 0 & \text{if } a=0 \\ \alpha_{a-1}(t)\frac{1-r_a}{1-r_{a-1}}V_{a-1,n} & \text{else} \end{cases}$$

$$\frac{dV_{a,r}}{dt} = \phi_{a,r}(t)S_{a,r} - \lambda_a(1-P)V_{a,r} - \alpha_a(t)V_{a,r} - \mu_a(t)V_{a,r} + \begin{cases} 0 & \text{if } a=0 \\ \alpha_{a-1}(t)\left(V_{a-1,r} + \frac{r_a - r_{a-1}}{1-r_{a-1}}V_{a-1,n}\right) & \text{else} \end{cases}$$

Individuals who are immune after infection *R*

$$\frac{dR_{a,n}}{dt} = \gamma I_{a,n} - \rho R_{a,n} - \alpha_a(t)R_{a,n} - \mu_a(t)R_{a,n} + \begin{cases} 0 & \text{if } a=0 \\ \alpha_{a-1}(t)\frac{1-r_a}{1-r_{a-1}}R_{a-1,n} & \text{else} \end{cases}$$

$$\frac{dR_{a,r}}{dt} = \gamma I_{a,r} - \rho R_{a,r} - \alpha_a(t)R_{a,r} - \mu_a(t)R_{a,r} + \begin{cases} 0 & \text{if } a=0 \\ \alpha_{a-1}(t)\left(R_{a-1,r} + \frac{r_a - r_{a-1}}{1-r_{a-1}}R_{a-1,n}\right) & \text{else} \end{cases}$$

Model parameters of Q-LAIV-Sim

Indexing

a	age group (in 6 month steps for the first two groups; in years thereafter)
n	category “no risk”
r	category “at risk”
t	time

Demographic parameters

$\nu(t)$	birth rate at time *t*
m	fraction of newborns with maternal protection
ω	rate at which maternal protection is lost
$\mu_a(t)$	death rate for age a and time *t*
r_a	fraction of age group a with “at risk” status
$\alpha_a(t)$	ageing rate of individuals of age a at time *t*

Natural history parameters

β_{a_S,a_I}	contact rate of susceptible individuals of age a_S with infectious individuals of age a_I
$\lambda_a(t)$	rate at which individuals of age a are infected at time *t*
δ	transition rate at which infected individuals in their latent stage become infectious
γ	rate at which infectious individuals recover and become immune
ρ	rate at which individuals who are immune because of infection lose their immunity

Vaccination parameters

$\phi_{a,n}(t)$	rate at which individuals of age a with status *n* (“no risk”) are vaccinated at time *t*
P	protection of successfully vaccinated individuals against infection

Table S1. Q-LAIV-Sim model parameters.

	Parameter	Age group	Value base case	Source
Population of Germany	Total size *N*	all ages	2015: 80.7 million<br>2025: 80.3 million	(3, 4)
	% at-risk r_a	18-29 years<br>30-44 years<br>45-59 years<br>60+ years	19.15%<br>28.65%<br>47.75%<br>100%	(5)
Transmission dynamics	Contact rate between individuals, β_{a_S,a_I}	all ages	extended POLYMOD matrix<br>'all reported contacts'	(6)
	Basic reproduction number R_0	all ages	1.1 (all-year average)	calibration, (7)
	Seasonality of transmission	all ages	peak on 21 Dec.<br>amplitude: 43% of baseline	(8)
	External infection introduction rate per person	all ages	baseline: 1/1,000 years, peak: Feb 8<br>amplitude: 100% of baseline	assumption
Natural histo-ry of influenza	Proportion of infected individuals developing symptoms	all ages	66.9%	(9)
	Duration of latency δ^{-1}	all ages	1 day	(9)
	Duration of contagiousness γ^{-1}	all ages	5 days	(9)
	Duration of immunity after infection ρ^{-1}	all ages	Influenza A: 6 years<br>Influenza B: 12 years	(8)

Table S2. Baseline vaccination coverage and vaccine efficacy used in Q-LAIV-Sim.
Vaccination takes place annually from 1 October to 30 November.

Age group	Baseline vaccination coverage	Vaccine efficacy	
		TIV	QLAIV
<1 year	0.3% (10)	45% (11)	---
1 year	2.8% (10)		
2 years	4.6% (10)	59% (11)	80% (12, 13)
3 years	5.4% (10)		
4 years	5.2% (10)		
5 years	5.3% (10)		
6 years	4.7% (10)		
7 years	5.3% (10)		
8 years	5.0% (10)		
9 years	4.8% (10)		
10 years	5.1% (10)		

Age group	Baseline vaccination coverage	Vaccine efficacy	
		TIV	QLAIV
11 years	5.3% (10)	59% (11)	80% (12, 13)
12 years	5.8% (10)		
13 years	5.3% (10)		
14 years	4.3% (10)		
15 years	4.0% (10)		
16 years	3.1% (10)		
17 years	2.8% (10)		
18-59, low risk	17.35% a) (14)	60% (15)	---
18-59, high risk	27.55% a) (14)	58% (16)	
60-69 years	43.3% a) (14)		
70-79 years	59.4% a) (14)		
80+ years	63.85% a) (14)		

a) average of the two reported seasons (2010/2011 and 2011/2012)

Further explanations of the mathematical model of Q-LAIV-Sim

Influenza strains

As influenza can be caused by any one of four different viral strains (A(H1N1), A(H3N2), B/Yamagata, B/Victoria), we assume that these four strains are transmitted independently, causing strain-specific immunity; i.e. the differential equations shown above have to be applied separately to each one of them. As trivalent vaccination (TIV) only contains one of the two B lineages, simulation results differ between strains.

Birth ν, death μ and ageing α

Using official demographic data until 2012 (3) and predicted data (using "Main scenario (proj_13npms)" from 2013 to 2026 (4), the simulation tool is constructed such that the exact size and age-distribution of the German population is reproduced for each simulation year. The numbers of individuals in the lowest age group are translated into birth rates ν and transitions from the numbers of individuals of age a in one year to age a+1 in the following year allow to calculate age-specific mortality rates μ_a. Unlike in other simulation tools (8, 17), births and deaths not only occur once a year, but are spread evenly over the simulation year. Whereas ageing in dynamic models frequently occurs only once a year (1, 8, 17), aging is implemented in Q-LAIV-Sim as a continuous process. Although the description of the differential equations seems to allow for individuals to continuously age, the simulation has been implemented such that each individual can only age once a year, using additional indexing of the differential equations which was omitted from the description. The only exception are the two age groups below 1 year of age: as influenza vaccination can only be performed for children of at least 6 months, newborn individuals also "celebrate" their 6-months anniversary instead of only having an annual "birthday" (i.e. they pass through two aging steps within one simulation year). As the percentage of individuals who are "at risk" when being infected (status *r*) increases over age, some individuals with "no risk" status (*n*) are added into the "at risk" category when they grow older. Individuals who reach the highest age group (100+ years) remain in that age-group until they die.

Maternal protection (fraction *m*, loss rate ω)

A fraction *m=30%* of newborn individuals is protected by maternal antibodies, the remaining fraction (1-*m*) is born susceptible. Maternal protection is lost at rate ω and the individuals become susceptible. As the average duration of maternal protection is assumed to be 4 months, only 0.25% of those newborns who initially are protected by maternal antibodies are still protected after 2 years. The model is implemented such that these few remaining individuals lose their maternal protection abruptly at their second birthday (this transition is not shown in the equations).

Contact matrix

Age-dependent mixing of contacts in Q-LAIV-Sim is based on the POLYMOD matrix for Germany (6). As Q-LAIV-Sim distinguishes annual cohorts (see above, paragraph "Birth ν, death μ and ageing α"), the original 5-year age group structure of the POLYMOD matrix was first extended into a 1-year structure, using a straight-forward extension algo-

rithm, and afterwards the resulting abrupt changes at the transitions between the former 5-year age groups were smoothed by rearranging contacts between cohorts such that the sum of contacts within the original 5-years age groups remained unchanged. As the POLYMOD matrix is by nature asymmetric, individuals of cohort a_1 may be predicted to have a total number of x contacts with individuals of cohort a_2, yet – viewed from the other side – individuals of cohort a_2 may be predicted to have a completely different number of contacts with cohort a_1. Such kinds of asymmetric contact structures may be appropriate for infectious diseases where the transmission is easier passed on from group a_1 to group a_2 than from a_2 to a_1 (as may be the case for the transmission of a venereal disease via bisexual contacts), yet such asymmetries should not play a major role in influenza transmission. In order to create a symmetric contact structure which is based on the POLYMOD study, we calculate an average of the (a_1,a_2) and the (a_2,a_1) contact rates which is weighted by the (time-varying) population sizes of the two cohorts. Performing this calculation for all 101 x 101 cohort combinations, we obtain a symmetric matrix $\beta_{a_1,a_2}(t)$ which we use to calculate the force of infection (cf. next paragraph).

Force of infection λ

To consider the seasonality of transmission, the force of infection depends on the time *t*, using the cosine function $(1+0.43\cdot\cos(2\pi(t-112)/365))$ which implies that infections are most effectively passed on around Christmas (112 days after the start of the simulation year, which is 1 September), reaching a value which is 43% higher than the all-year average (8). This seasonality function is multiplied by the sum of all infectious individuals $\sum_{a_I}\left(I_{a_I,n}(t)+I_{a_I,r}(t)\right)$.

Age-dependent contacts are governed by an extension of the POLYMOD contact matrix (6) to one-year age classes (see previous paragraph for details), leading to the contact rate β_{a_S,a_I} of infected individuals of age a_I with susceptible individuals of age a_S . Furthermore, infectious contacts can also occur with individuals from outside of the population ("external" or "imported" infections) which also depends on the time of the year, peaking in February; the external infection rate is given by $(1+\cos(2\pi(t-160)/365))\cdot 0.001$, assuming that individuals are infected from outside at a rate of 1 per 1,000 years. Putting everything together leads to the time-dependent infection rate of individuals of age a_S:

$$\lambda_{a_S}(t)=\left(\left(1+0.43\cdot\cos\left(2\pi\frac{t-112}{365}\right)\right)\cdot\sum_{a_I}\frac{\beta_{a_S,a_I}(t)}{N_{a_S}(t)}\left(I_{a_I,n}(t)+I_{a_I,r}(t)\right)\right)+\left(1+\cos\left(2\pi\frac{t-160}{365}\right)\right)\cdot 0.001$$

Vaccination rate ϕ

Vaccinations are implemented such that, in a given simulation year approximately the same number of vaccinations takes place on every day during October and November, and that finally the given age- and risk-specific vaccination coverage is reached (for a mathematical description, see (1)). Vaccinations depend on the age and the risk status of the individuals, but not on their immunity status. As vaccination of immune individuals and infected individuals is assumed not to change their immunologic status, these vaccinations are not shown in the equations above, but they are

recorded during the simulation, using additional indexing of the differential equations. This is necessary for two reasons: (a) although the description of the differential equations above would allow for individuals to receive multiple vaccinations per year, the system is implemented such that each individual can only be vaccinated once a year. (b) individuals who were vaccinated in the previous year are assumed to preferentially be re-vaccinated in the next year (an estimation of the re-vaccination factor for Germany is given below). Only the vaccination of susceptible individuals can change their immunity status, depending on the vaccine efficacy (which depends on the age and risk status of the vaccinee and on the type of vaccine). Whereas only tetravalent vaccines (QIV and QLAIV) are used during the evaluation period of the simulations (starting in 2017), trivalent inactivated vaccine (TIV) is used during the initialization period (2000-2016). TIV can protect against both Influenza A strains, but only contains one of the two Influenza B lineages which changes from year to year, following the recommendation of the WHO. The recorded TIV composition is used in the simulation tool, and the vaccine efficacy is set to zero for the B lineage which is missing in TIV in the respective year.

Protection *P* of vaccinees against infection

In the standard version of the simulation tool Q-LAIV-Sim, successfully vaccinated individuals are fully protected against infection and disease until they lose their immunity (loss of vaccination-derived immunity will be described in the next section). In order to reproduce the reasoning which is given above for the SIR and SIRS models, a new parameter *P* was introduced which describes the protection of vaccinees against infection. With the default value *P*=1, vaccinees cannot be infected until they lose their immunity; with the experimental value *P*=0, vaccinees remain fully susceptible and the number of vaccinated individuals who are infected while they are still in category "*V*" can be calculated (in order not to change anything during the initialization procedure or in the reference scenario with QIV, the setting *P*=0 is only used for QLAIV vaccination).

Loss rate of vaccine-derived immunity (not shown in equations)

Vaccinations with the inactivated vaccines (TIV and QIV) are assumed to protect the vaccinees for one season. Individuals who have become immune due to TIV or QIV vaccination lose their immunity at the end of the simulation year (i.e. on 31 August) in which they were vaccinated. For the live vaccine, it has been shown that a percentage of individuals are still protected in the second season: the vaccine efficacy in the first season was 80% (12, 13); it dropped to 56% in the second year (18), i.e. 70% of the individuals who are protected in the first year, are also protected in the second year. The model is implemented such that 30% of individuals lose their Q-LAIV immunity at the end of the simulation year in which they are vaccinated, and the remaining ones lose their immunity at the end of the following year. Technically, this demands further indexing of the differential equations which is not shown in the model description.

Simulation results of Q-LAIV-Sim

Simulation results are given in Table S3 and in Table 1 and Figure 5 of the paper. In a sensitivity analysis, we have used the QIV vaccine efficacy and the duration of QIV immune protection of QLAIV, too (Table S4).

Each simulations was run three times:

a) Baseline vaccination coverage (i.e. no additional vaccinations), using QIV vaccine
b) like in (a), but children receive Q-LAIV vaccine with a higher vaccination coverage (fully protective vaccine)
c) like in (b), but using a completely non-protective vaccine

Effects were calculated as follows:

All prevented infections (children) a_C	10 year difference in cumulative infection incidence for susceptible children (i.e. children in stage "S"): simulation (a) minus simulation (b)
Directly prevented infections (children) d_C	10 year cumulative infection incidence for vaccinees (i.e. children in stage "V"): using simulation (c)
Indirectly prevented infections (children) i_C	a_C - d_C
Indirect/direct ratio (children) r_C	i_C/d_C
Indirectly prevented infections (adults) i_A	10 year difference in cumulative infection incidence for susceptible adults (i.e. adults in stage "S"): simulation (a) minus simulation (b)
Indirectly prevented infections (total) i_T	$i_C + i_A$
Indirect/direct ratio (total) r_T	i_T/d_C

Table S3. Annual number of directly and indirectly prevented influenza-related events caused by pediatric QLAIV vaccination in Germany. Simulations are initialized from 2010 to 2016 using TIV with the baseline vaccination coverage shown in Table S2. In the reference scenario, TIV is replaced by QIV in 2017, but the vaccination coverage remains unchanged. In the evaluated scenario, the same QIV vaccinations are performed, except for 2-17 year old children who receive QLAIV; in the first evaluation year their QLAIV coverage is identical to the baseline coverage (around 5%), then it is increased in three equal annual steps to reach the final coverage as shown below. In the columns "Prevented cases", 10-year cumulative numbers of directly (d_C) and indirectly prevented cases among children (i_C), as well as indirectly prevented cases among adults (i_A) and in the total population (i_T) are given. In the "Ratio" columns, ratios of indirectly prevented cases divided by directly prevented cases are given for the sub-group of children (r_C) and for the total population (r_T). For a graphical display of the results see Figure 5 of the paper.

QLAIV (+) coverage	Symptomatic cases		Acute otitis media (AOM)		Deaths	
	Prevented cases	Ratio	Prevented cases	Ratio	Prevented cases	Ratio
20	d_C: 1,080,655.0<br>i_C: 2,408,791.0<br>i_A: 5,535,926.1<br>i_T: 7,944,717	r_C: 2.2<br>r_A: ----<br>r_T: 7.4	d_C: 71,477.3<br>i_C: 253,150.5<br>i_A: 55,359.2<br>i_T: 308,509.8	r_C: 3.5<br>r_A: ----<br>r_T: 4.3	d_C: 111.9<br>i_C: 397.8<br>i_A: 3,624.9<br>i_T: 4,022.4	r_C: 3.6<br>r_A: ----<br>r_T: 35.9
25	d_C: 1,427,175.0<br>i_C: 2,751,614.7<br>i_A: 6,815,377.2<br>i_T: 956,6992.1	r_C: 1.9<br>r_A: ----<br>r_T: 6.7	d_C: 98,020.3<br>i_C: 292,409.6<br>i_A: 68,153.5<br>i_T: 360,563.1	r_C: 3.0<br>r_A: ----<br>r_T: 3.7	d_C: 153.6<br>i_C: 459.0<br>i_A: 4,458.9<br>i_T: 4,917.7	r_C:3.0<br>r_A: ----<br>r_T: 32.0
30	d_C: 1,765,551.0<br>i_C: 3,023,809.4<br>i_A: 8,018,576.8<br>i_T: 11,042,386.3	r_C: 1.7<br>r_A: ----<br>r_T: 6.3	d_C: 123,988.0<br>i_C: 325,399.9<br>i_A: 80,185.6<br>i_T: 405,585.6	r_C: 2.6<br>r_A: ----<br>r_T: 3.3	d_C: 194.4<br>i_C: 510.6<br>i_A: 899.4<br>i_T: 5,752.2	r_C: 2.6<br>r_A: ----<br>r_T: 29.6
35	d_C: 2,098,649.0<br>i_C: 3,232,049.8<br>i_A: 9,148,224.1<br>i_T: 12,380,273.8	r_C: 1.5<br>r_A: ----<br>r_T: 5.9	d_C: 149,673.0<br>i_C: 352,707.5<br>i_A: 91,482.1<br>i_T: 444,189.8	r_C: 2.4<br>r_A: ----<br>r_T: 3.0	d_C: 234.7<br>i_C: 552.9<br>i_A: 5,976.0<br>i_T: 6,528.7	r_C: 2.4<br>r_A: ----<br>r_T: 27.8
40	d_C: 2,427,139.0<br>i_C: 3,381,844.2<br>i_A: 10,202,766.7<br>i_T: 13,584,610.9	r_C: 1.4<br>r_A: ----<br>r_T: 5.6	d_C: 175,026.9<br>i_C: 374,763.3<br>i_A: 102,027.5<br>i_T: 476,790.9	r_C: 2.1<br>r_A: ----<br>r_T: 2.7	d_C: 274.6<br>i_C: 586.8<br>i_A: 6,660.4<br>i_T: 7,247.0	r_C: 2.1<br>r_A: ----<br>r_T: 26.4
45	d_C: 2,752,099.0<br>i_C: 3,478,910.0<br>i_A: 11,183,493.3<br>i_T: 14,662,403.4	r_C: 1.3<br>r_A: ----<br>r_T: 5.3	d_C: 200,134.2<br>i_C: 392,038.4<br>i_A: 111,834.6<br>i_T: 503,873.4	r_C:2.0<br>r_A: ----<br>r_T: 2.5	d_C: 314.0<br>i_C: 613.4<br>i_A: 7,296.3<br>i_T: 7,909.7	r_C: 2.0<br>r_A: ----<br>r_T: 25.2
50	d_C: 3,074,578.0<br>i_C: 3,528,638.1<br>i_A: 12,092,827.6<br>i_T: 15,621,465.6	r_C: 1.1<br>r_A: ----<br>r_T: 5.1	d_C: 225,100.2<br>i_C: 404,967.7<br>i_A: 120,928.0<br>i_T: 525,895.9	r_C:1.8<br>r_A: ----<br>r_T: 2.3	d_C: 353.2<br>i_C: 633.2<br>i_A: 7,885.4<br>i_T: 8,518.6	r_C: 1.8<br>r_A: ----<br>r_T: 24.1
55	d_C: 3,400,403.0<br>i_C: 3,533,613.6<br>i_A: 12,938,352.6<br>i_T: 16,471,966.2	r_C: 1.0<br>r_A: ----<br>r_T: 4.8	d_C: 250,320.0<br>i_C: 413,873.5<br>i_A: 129,383.2<br>i_T: 543,256.9	r_C: 1.7<br>r_A: ----<br>r_T: 2.2	d_C: 392.8<br>i_C: 646.7<br>i_A: 8,432.6<br>i_T: 9,079.2	r_C: 1.6<br>r_A: ----<br>r_T: 23.1
60	d_C: 3,725,882.0<br>i_C: 3,500,241.9<br>i_A: 13,719,026.5<br>i_T: 17,219,268.4	r_C: 0.9<br>r_A: ----<br>r_T: 4.6	d_C: 275,545.6<br>i_C: 419,200.0<br>i_A: 137,190.2<br>i_T: 556,390.2	r_C: 1.5<br>r_A: ----<br>r_T: 2.0	d_C: 432.2<br>i_C: 654.7<br>i_A: 8,937.4<br>i_T: 9,592.2	r_C: 1.5<br>r_A: ----<br>r_T: 22.2

Table S4. Annual number of directly and indirectly prevented influenza-related events caused by pediatric QLAIV vaccination in Germany. In these simulations, the vaccine efficacy of QLAIV was set to the QIV value of 59% and the duration of QLAIV immunity was set to one year (as for QIV); all other details are described in Table S3.

QLAIV (-) coverage	Symptomatic cases		Acute otitis media (AOM)		Deaths	
	Prevented cases	Ratio	Prevented cases	Ratio	Prevented cases	Ratio
20	d_C: 378,140.3<br>i_C: 1,361,036.4<br>i_A: 2,448,750.7<br>i_T: 3,809,787.2	r_C: 3.6<br>r_A: ----<br>r_T: 10.1	d_C: 19,214.4<br>i_C: 141,048.1<br>i_A: 24,487.4<br>i_T: 165,535.7	r_C: 7.3<br>r_A: ----<br>r_T: 8.6	d_C: 29.1<br>i_C: 222.4<br>i_A: 1,611.7<br>i_T: 1,833.9	r_C: 7.6<br>r_A: ----<br>r_T: 63.0
25	d_C: 613,314.5<br>i_C: 1,648,221.3<br>i_A: 3,235,391.1<br>i_T: 4,883,612.5	r_C: 2.7<br>r_A: ----<br>r_T: 8.0	d_C: 37,437.2<br>i_C: 171,483.8<br>i_A: 32,353.8<br>i_T: 203,837.9	r_C: 4.6<br>r_A: ----<br>r_T: 5.4	d_C: 57.7<br>i_C: 270.1<br>i_A: 2,128.3<br>i_T: 2,438.2	r_C: 4.7<br>r_A: ----<br>r_T: 42.3
30	d_C: 848,376.7<br>i_C: 1,913,038.1<br>i_A: 4,017,882.2<br>i_T: 5,930,920.3	r_C: 2.3<br>r_A: ----<br>r_T: 7.0	d_C: 55,649.8<br>i_C: 200,029.7<br>i_A: 40,178.6<br>i_T: 240,208.4	r_C: 3.6<br>r_A: ----<br>r_T: 4.3	d_C: 86.3<br>i_C: 315.2<br>i_A: 2,641.4<br>i_T: 2,956.3	r_C: 3.7<br>r_A: ----<br>r_T: 34.3
35	d_C: 1,083,510.1<br>i_C: 2,154,230.2<br>i_A: 4,793,013.1<br>i_T: 6,947,243.1	r_C: 2.0<br>r_A: ----<br>r_T: 6.4	d_C: 73,887.1<br>i_C: 226,586.9<br>i_A: 47,930.0<br>i_T: 274,517.0	r_C: 3.1<br>r_A: ----<br>r_T: 3.7	d_C: 114.9<br>i_C: 356.7<br>i_A: 3,149.3<br>i_T: 3,505.9	r_C: 3.1<br>r_A: ----<br>r_T: 30.5
40	d_C: 1,318,571.1<br>i_C: 2,370,769.6<br>i_A: 5,557,010.1<br>i_T: 7,927,779.8	r_C: 1.8<br>r_A: ----<br>r_T: 6.0	d_C: 92,119.2<br>i_C: 251,058.2<br>i_A: 55,570.0<br>i_T: 306,628.5	r_C: 2.7<br>r_A: ----<br>r_T: 3.3	d_C: 143.5<br>i_C: 394.9<br>i_A: 3,649.1<br>i_T: 4,044.1	r_C: 2.9<br>r_A: ----<br>r_T: 28.2
45	d_C: 1,553,593.6<br>i_C: 2,562,164.6<br>i_A: 6,306,854.0<br>i_T: 8,869,018.7	r_C: 1.6<br>r_A: ----<br>r_T: 5.7	d_C: 110,353.3<br>i_C: 273,395.1<br>i_A: 63,068.5<br>i_T: 336,463.8	r_C:2.5<br>r_A: ----<br>r_T: 3.0	d_C: 172.2<br>i_C: 429.8<br>i_A: 4,139.2<br>i_T: 4,568.9	r_C: 2.5<br>r_A: ----<br>r_T: 26.5
50	d_C: 1,788,642.0<br>i_C: 2,728,270.8<br>i_A: 7,039,884.8<br>i_T: 9,768,155.6	r_C: 1.5<br>r_A: ----<br>r_T: 5.5	d_C: 128,602.6<br>i_C: 293,574.7<br>i_A: 70,398.7<br>i_T: 363,973.6	r_C: 2.3<br>r_A: ----<br>r_T: 2.8	d_C: 200.8<br>i_C: 461.5<br>i_A: 4,617.6<br>i_T: 5,078.8	r_C: 2.3<br>r_A: ----<br>r_T: 25.3
55	d_C: 2,023,748.5<br>i_C: 2,868,948.4<br>i_A: 7,753,282.1<br>i_T: 10,622,230.4	r_C: 1.4<br>r_A: ----<br>r_T: 5.2	d_C: 146,872.4<br>i_C: 311,574.6<br>i_A: 77,532.7<br>i_T: 389,107.6	r_C: 2.1<br>r_A: ----<br>r_T: 2.6	d_C: 229.2<br>i_C: 489.6<br>i_A: 5,082.7<br>i_T: 5,572.2	r_C: 2.1<br>r_A: ----<br>r_T: 24.3
60	d_C: 2,258,920.5<br>i_C: 2,984,960.3<br>i_A: 8,445,515.0<br>i_T: 11,430,475.1	r_C: 1.3<br>r_A: ----<br>r_T: 5.1	d_C: 165,164.3<br>i_C: 327,444.3<br>i_A: 84,455.0<br>i_T: 411,899.7	r_C: 2.0<br>r_A: ----<br>r_T: 2.5	d_C: 258.1<br>i_C: 514.0<br>i_A: 5,533.6<br>i_T: 6,047.5	r_C: 2.0<br>r_A: ----<br>r_T: 23.4

Estimation of the re-vaccination factor

The preferential re-vaccination factor will be calculated from reported data on people who were vaccinated at least once in three subsequent seasons from 2004/5 to 2006/7 in Germany (Table S4). Vaccination coverage of >60 year olds was 2004/5: p_1=45%, 2005/6: p_2=50% and 2006/7: p_3=49%, respectively; the vaccination coverage p_0 below 60 years was not reported (19).

Table S4. Number of individuals who received one, two or three vaccinations in Germany during the seasons 2004/5, 2005/6 and 2006/7 (19).

age group	Reported number of vaccinations	Reported number of individuals	Reported percentage of vaccinees of the age group	Estimated percentage, using a re-vaccination factor *f*=4.25
≤ 60 years	1	3.688.367	50.3%	48.8%
	2	1.998.431	27.2%	30.8%
	3	1.649.990	22.5%	20.5%
> 60 years	1	2.198.497	24.2%	24.1%
	2	2.583.421	28.3%	28.0%
	3	4.308.334	47.4%	47.9%

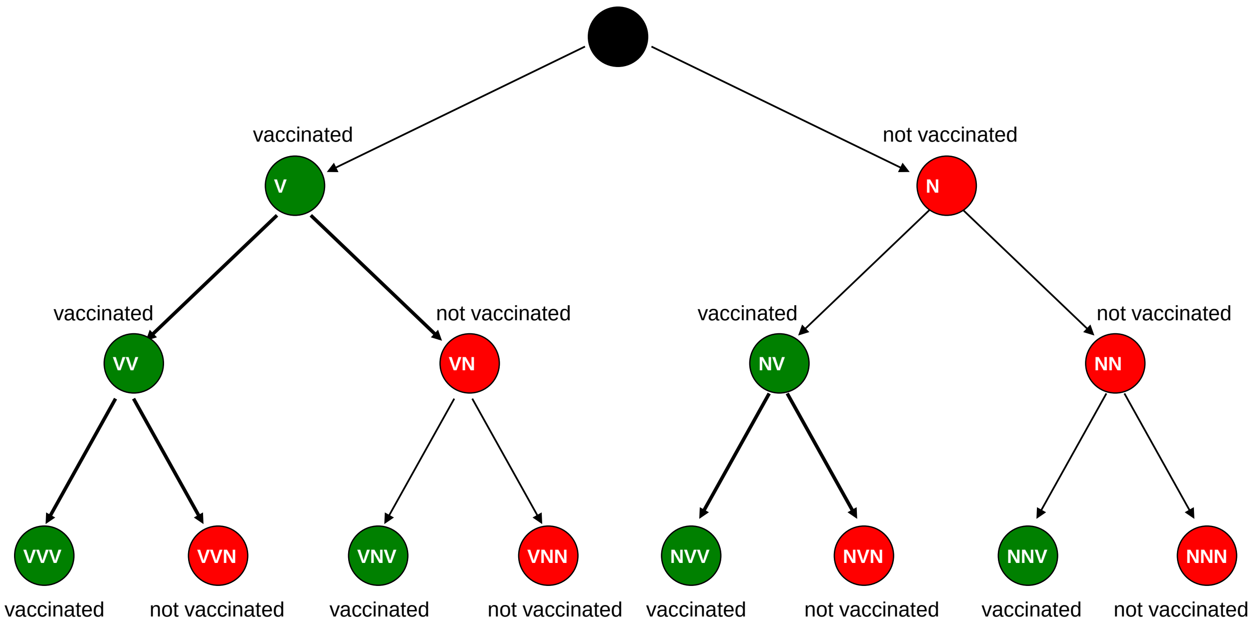

Figure S1. Visualization of vaccination sequences of individuals who made three vaccination choices.

Expected percentage of people >60 years who were vaccinated once, twice or three times

As percentages p_1, p_2 and p_3 of individuals >60 years were vaccinated in 2004/5, 2005/6 and 2006/7, respectively, the expected percentages of people who received one, two or three vaccinations can be calculated if we assume that individuals who were vaccinated in the previous year are *f* times as likely to be vaccinated again in the current year as people who were not vaccinated in the previous year; abbreviations are explained by the decision tree depicted in Figure S1.

2004/5: $V = p_1$, $N = 1 - p_1$

2005/6: $VV = V \cdot fc_2$, $VN = V(1 - fc_2)$, $NV = N \cdot c_2$, $NN = N(1 - c_2)$ with $c_2 = \dfrac{p_2}{Vf + N}$

2006/7: $VVV = VV \cdot fc_3$, $VVN = VV(1 - fc_3)$, $VNV = VN \cdot c_3$, $VNN = VN(1 - c_3)$, $NVV = NV \cdot fc_3$,

$NVN = NV(1 - fc_3)$, $NNV = NN \cdot c_3$, $NNN = NN(1 - c_3)$ with $c_3 = \dfrac{p_3}{(VV + NV)f + (VN + NN)}$

Expected percentage of people ≤60 years who were vaccinated once, twice or three times

As the percentages of individuals ≤60 years who were vaccinated in 2004/5, 2005/6 and 2006/7, respectively, were not given, we assume that an (unknown) percentage p_0 of them were vaccinated in every single year. Using this percentage (which will be estimated together with the revaccination factor *f*), we can again calculate the expected percentages of individuals who received one, two or three vaccinations:

2004/5: $V = p_0$, $N = 1 - p_0$

2005/6: $VV = V \cdot fc_2$, $VN = V(1 - fc_2)$, $NV = N \cdot c_2$, $NV = N(1 - c_2)$, $NN = N \cdot c_2$ with $c_2 = \dfrac{p_0}{Vf + N}$

2006/7: $VVV = VV \cdot fc_3$, $VVN = VV(1 - fc_3)$, $VNV = VN \cdot c_3$, $VNN = VN(1 - c_3)$, $NVV = NV \cdot fc_3$,

$NVN = VN(1 - fc_3)$, $NNV = NN \cdot c_3$, $NNN = NN(1 - c_3)$ with $c_3 = \dfrac{p_0}{(VV + NV)f + (VN + NN)}$

Estimation of the re-vaccination factor *f*

As the data set only contains individuals who were vaccinated at least once, we obtain that

- a fraction $(VNN + NVN + NNV)/(1 - NNN)$ is expected to be vaccinated once
- a fraction $(VVN + VNV + NVV)/(1 - NNN)$ is expected to be vaccinated twice
- a fraction $VVV/(1 - NNN)$ is expected to be vaccinated three times

The unknown parameters p_0 and *f* were estimated using the method of least squares, obtaining the following estimates:

- p_0 = 25.9% of young adults were vaccinated
- *f* = 4.25, meaning that previously vaccinated individuals are more than 4 times as likely to be vaccinated again as previously unvaccinated individuals

As can be seen from the two last columns of Table S4, the estimated percentages of individuals with one, two or three vaccinations (using *f*=4.25) are satisfactorily close to the observed ones.

Exploring direct and indirect vaccination effects in a static model with seasonality

The simulation results in Q-LAIV-Sim are strongly driven by the seasonality of transmission and by the annual vaccination campaigns which precede the transmission seasons (Figure S2). Seasonality was not incorporated in the SIR or the SIRS models which were explored above, but we have developed a simple static model which captures the most important features of the seasonal changes, using the sinusoidal function of Vynnycky *et al*. 2008 (8) according to which transmission is 43% higher around Christmas than on average.

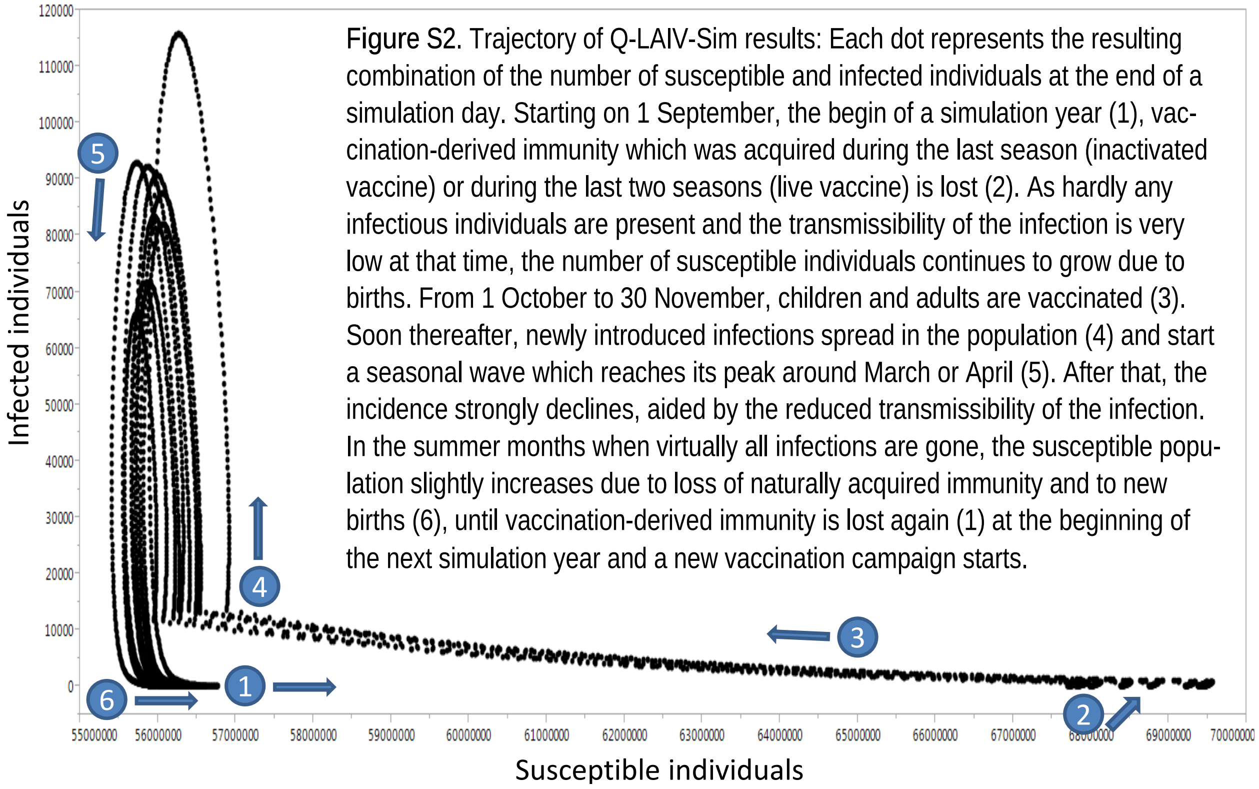

Figure S2. Trajectory of Q-LAIV-Sim results: Each dot represents the resulting combination of the number of susceptible and infected individuals at the end of a simulation day. Starting on 1 September, the begin of a simulation year (1), vaccination-derived immunity which was acquired during the last season (inactivated vaccine) or during the last two seasons (live vaccine) is lost (2). As hardly any infectious individuals are present and the transmissibility of the infection is very low at that time, the number of susceptible individuals continues to grow due to births. From 1 October to 30 November, children and adults are vaccinated (3). Soon thereafter, newly introduced infections spread in the population (4) and start a seasonal wave which reaches its peak around March or April (5). After that, the incidence strongly declines, aided by the reduced transmissibility of the infection. In the summer months when virtually all infections are gone, the susceptible population slightly increases due to loss of naturally acquired immunity and to new births (6), until vaccination-derived immunity is lost again (1) at the beginning of the next simulation year and a new vaccination campaign starts.

Using an all-year average basic reproduction number R_0=1.1 and the seasonality function of Vynnycky *et al*., we get the following seasonal fluctuations of the reproduction number: $R_0(t) = 1.1 \cdot (1 + 0.43 \cdot \cos(2\pi \cdot (t-112)/365))$. As annually only 10.6% of individuals are infected with influenza (7), only about 2-3% of the population is infected with any one of the four influenza strains. In simulations with Q-LAIV-Sim (using the parameter settings shown in Tables S1 and S2), about 30% of the population are immune to a given influenza strain before the transmission season. Because of the low infection rate, this percentage hardly changes during the transmission season (as can be seen from Figure S2, stages 1-3, vaccination and loss of vaccination-derived immunity which take place outside of the seasonal transmission window have much more impact on the number of susceptibles). As the number of immune individuals only slightly changes during the transmission period, seasonality must be a major driver of the dynamics which determines the increase and decrease in the number of cases.

In the following simplified model, we assume that the immunity does not change during the seasonal wave at all, but remains at a constant level of 30%. We start with a single infection on 1 December (i.e. $t_0 = 91$, which is immediately after the end of the annual vaccination campaign and which precedes the seasonal wave). At this time, the value of the reproduction number $R(t_0)$ is 1.542, meaning that the index case is expected to infect 1.542 others in a non-immune population. As 30% of the population is immune, the expected number of secondary infections is reduced to 1.08 which is just above the critical number 1.0 which is needed for an epidemic to evolve. On average, it takes a generation time of 3.5 days until the next generation of cases is ready to infect others. By then, the reproduction number has increased to $R(t_0 + 3.5) = 1.552$, and each of the expected 1.08 cases infects 1.09 others, bringing the expected number to 1.173. This continues with changing R_0 values so that a seasonal influenza wave builds up. As transmission eventually passes its peak, the expected number of new cases will finally decline and the seasonal wave will end. The expected number of cases in the k-th transmission generation (each of which takes on average 3.5 days) can be calculated as $\prod_{i=1}^{k}(R_k \cdot (1-0.3))$ whereby $R_k = R_0(t_0 + 3.5(k-1))$. Summed up over the whole year, introduction of one case on 1 December finally leads to 89.0 cases. Vaccination can easily be incorporated in this formula: if 2% of the population who are still susceptible at the end of 30 November are (additionally) vaccinated before introduction of the index case, the formula needs to be changed to $\prod_{i=1}^{k}(R_k \cdot (1-0.3) \cdot (1-0.02))$. Although this seems to negligibly decrease the slope at which the number of cases grows, the expected annual number of cases drops from 89.0 to 62.5 cases (i.e. by 29.2%). As only 2% of the population were vaccinated, only 2% of the original 89.0 secondary cases (i.e. 1.8 cases) were prevented because the prospective victims were protected (i.e. directly protected). The remaining 24.8 cases which were prevented by vaccinating 2% of the population were obviously an indirect effect of vaccination. The ratio of indirectly/directly prevented cases was, thus 24.8/1.8 = 13,8, meaning that more than nearly 14 times as many cases were prevented indirectly as were prevented directly.

References

1. Rose MA, Damm O, Greiner W, Knuf M, Wutzler P, Liese JG, et al. The epidemiological impact of childhood influenza vaccination using live-attenuated influenza vaccine (LAIV) in Germany: predictions of a simulation study. BMC Infect Dis. 2014;14:40.
2. Damm O, Eichner M, Rose MA, Knuf M, Wutzler P, Liese JG, et al. Public health impact and cost-effectiveness of intranasal live attenuated influenza vaccination of children in Germany. Eur J Health Econ. 2015;16(5):471-88.
3. Statistisches Bundesamt. Bevölkerung Deutschlands bis 2060. 12. koordinierte Bevölkerungsvorausberechnung. 2009.
4. European Commission. 2014 [Available from: http://epp.eurostat.ec.europa.eu/portal/page/portal/eurostat/home/.
5. Fleming DM, Elliot AJ. Estimating the risk population in relation to influenza vaccination policy. Vaccine. 2006;24(20):4378-85.
6. Mossong J, Hens N, Jit M, Beutels P, Auranen K, Mikolajczyk R, et al. Social contacts and mixing patterns relevant to the spread of infectious diseases. PLoS Med. 2008;5(3):e74.
7. Williams CJ, Schweiger B, Diner G, Gerlach F, Haaman F, Krause G, et al. Seasonal influenza risk in hospital healthcare workers is more strongly associated with household than occupational exposures: results from a prospective cohort study in Berlin, Germany, 2006/07. BMC Infect Dis.10:8.
8. Vynnycky E, Pitman R, Siddiqui R, Gay N, Edmunds WJ. Estimating the impact of childhood influenza vaccination programmes in England and Wales. Vaccine. 2008;26(41):5321-30.
9. Carrat F, Vergu E, Ferguson NM, Lemaitre M, Cauchemez S, Leach S, et al. Time lines of infection and disease in human influenza: a review of volunteer challenge studies. Am J Epidemiol. 2008;167(7):775-85.
10. Schröder-Bernhardi D, Grunow SS, Bauman U, Zöllner Y. Schutzimpfungen gegen Influenza werden bei Kindern und Jugendlichen äußerst selten durchgeführt – Ergebnisse einer Datenbankanalyse. 9 Jahrestagung der Deutschen Gesellschaft für Epidemiologie; 17-20 Sept 2014; Ulm2014.
11. Jefferson T, Rivetti A, Di Pietrantonj C, Demicheli V, Ferroni E. Vaccines for preventing influenza in healthy children. Cochrane Database Syst Rev. 2012(8):CD004879.
12. Jefferson T, Rivetti A, Harnden A, Di Pietrantonj C, Demicheli V. Vaccines for preventing influenza in healthy children. Cochrane Database Syst Rev. 2008(2):CD004879.
13. Rhorer J, Ambrose CS, Dickinson S, Hamilton H, Oleka NA, Malinoski FJ, et al. Efficacy of live attenuated influenza vaccine in children: A meta-analysis of nine randomized clinical trials. Vaccine. 2009;27(7):1101-10.
14. Bodeker B, Remschmidt C, Muters S, Wichmann O. [Influenza, tetanus, and pertussis vaccination coverage among adults in Germany]. Bundesgesundheitsblatt Gesundheitsforschung Gesundheitsschutz. 2015;58(2):174-81.
15. Jefferson T, Di Pietrantonj C, Rivetti A, Bawazeer GA, Al-Ansary LA, Ferroni E. Vaccines for preventing influenza in healthy adults. Cochrane Database Syst Rev. 2014(7):CD001269.
16. Jefferson T, Di Pietrantonj C, Al-Ansary LA, Ferroni E, Thorning S, Thomas RE. Vaccines for preventing influenza in the elderly. Cochrane Database Syst Rev. 2010(2):CD004876.
17. Pitman RJ, Nagy LD, Sculpher MJ. Cost-effectiveness of childhood influenza vaccination in England and Wales: Results from a dynamic transmission model. Vaccine.31(6):927-42.
18. Tam JS, Capeding MR, Lum LC, Chotpitayasunondh T, Jiang Z, Huang LM, et al. Efficacy and safety of a live attenuated, cold-adapted influenza vaccine, trivalent against culture-confirmed influenza in young children in Asia. Pediatr Infect Dis J. 2007;26(7):619-28.
19. Reuss AM, Walter D, Feig M, Kappelmayer L, Buchholz U, Eckmanns T, et al. Influenza vaccination coverage in the 2004/05, 2005/06, and 2006/07 seasons: a secondary data analysis based on billing data of the German associations of statutory health insurance physicians. Dtsch Arztebl Int. 2010;107(48):845-50.
